# Exploring the mediating role of depression and anxiety in the relationship between social and biological factors and quality of life in Indonesia: a structural equation modelling approach

**DOI:** 10.1136/bmjopen-2024-095110

**Published:** 2025-04-28

**Authors:** Herni Susanti, Mashita Fajri, Budi Anna Keliat, Helen Brooks, Penny Bee, Asri Maharani

**Affiliations:** 1 Mental Health Nursing, Faculty of Nursing, Universitas Indonesia, Depok, Indonesia; 2 Mental Health Research Group, Division of Nursing, Midwifery and Social Work, School of Health Sciences, Faculty of Biology, Medicine, and Health and Manchester Academic Health Science Centre, The University of Manchester, Manchester, UK

**Keywords:** MENTAL HEALTH, Quality of Life, Anxiety disorders, Depression & mood disorders

## Abstract

**ABSTRACT:**

**Objectives:**

To examine the association between social and biological factors and quality of life (QoL), and whether depression and anxiety mediate this relationship.

**Design:**

Cross-sectional study with individual level as the unit of analysis.

**Main outcome measures:**

Depression and anxiety were measured using the Center for Epidemiologic Studies Short Depression scale and Generalized Anxiety Disorder scale, while QoL was assessed using the EuroQol Five-Dimension scale. Social factors were assessed using the self-reported number of close persons, and biological factors were measured using the number of self-reported physical health comorbidities.

**Setting:**

Country-level data.

**Participants:**

General population aged 18 and older with data available.

**Results:**

Among participants, 849 (4.42%) had depression and 2339 (12.17%) had anxiety. Structural equation modelling (SEM) analysis, adjusted by age and sex, showed that social factors (β=−0.004, p<0.001) and biological factors (β=−0.051, p<0.001) were directly associated with QoL. Mediation analysis revealed that depression and anxiety fully mediated the relationship between social factors and QoL. For biological factors, depression and anxiety partially mediated the relationship with QoL, accounting for 29.30% and 22.83% of the total effect, respectively.

**Conclusions:**

Depression and anxiety mediate the relationship between social and biological factors and QoL. Strengthening social support and improving mental health access can mitigate these risks. Future research should examine long-term trends and intervention effectiveness to inform targeted policies for at-risk populations.

**STRENGTHS AND LIMITATIONS OF THIS STUDY**

- We used a large population-based sample of over 19 000 participants from 12 villages across four provinces in Java, selected to reflect urban and rural diversity.
- A structured random sampling process was conducted at the neighbourhood (*rukun tetangga or RT*) level to minimise selection bias.
- Depression and anxiety were assessed using Center for Epidemiologic Studies Short Depression (CES-D-10) scale and Generalised Anxiety Disorder scale, which have been validated in Indonesia, although internal consistency for CES-D-10 was modest.
- Structural equation modelling was applied to explore complex mediation pathways between social and biological factors, mental health and quality of life.
- The cross-sectional design and self-reported comorbidity data limit causal inference and may introduce recall or reporting bias.

## Background

 Mental health disorders are among the most significant global health challenges. In 2019, it was reported that approximately 970 million individuals worldwide, equating to 1 in every 8 people, were experiencing various mental problems, with anxiety and depression standing out as the most prevalent conditions.^1^ Over 80% of individuals with mental problems reside in low- and middle-income countries (LMICs), where the interplay between mental health and poverty is particularly severe due to inadequate welfare systems and limited access to effective treatment.^2^ In Indonesia, more than nine million people suffer from depression and anxiety, making it one of the top two countries in the WHO’s South-East Asia region for prevalence.^3^ With estimated annual treatment costs per patient for depression and anxiety ranging from $85 to $114, a recent study in Indonesia estimated the total national cost for these mental health issues could reach up to $6.244 billion.^4^

Depression and anxiety are complex conditions with multiple causes, including social and biological factors. Social factors are crucial to mental health, as shown by two systematic reviews that found a strong link between protective social factors and a lower likelihood of depression,^5^ as well as reduced severity of anxiety.^6^ Biological factors also significantly affect quality of life (QoL), as poor self-rated health, abnormal body mass index and the presence of chronic illnesses have been strongly associated with higher levels of depression^7^ and anxiety.^8^ Furthermore, prior studies have shown that both social and biological factors affect QoL.^9 10^ For example, a study in Ethiopia found that social support level and duration of illness were significant predictors of QoL.^9^ Additionally, a study conducted in Iran, another LMIC, also found that higher perceived social support and not having any chronic diseases were associated with higher QoL.^10^ These relationships suggest that social and biological factors and QoL may be influenced either directly or indirectly by the presence of depression and anxiety.

Depression and anxiety significantly impact individuals across all stages of life. In adults, particularly, those with depression or anxiety experience a 20% increase in presenteeism, work productivity impairment and activity impairment, leading to cost-savings estimates between $14 000 and more than $18 000 annually.^11^ Prior studies have shown that untreated depression and anxiety reduce the overall QoL of an individual.^12^,^14^ For example, a Hungarian study found that depressed men had lower QoL scores compared with non-depressed men (47.38 vs 50.19 respectively).^12^ Additionally, research shows that more severe anxiety symptoms are closely associated with poorer psychological and physical QoL compared with those without an anxiety disorder, regardless of the specific anxiety type.^13^ Research in LMICs shows similar trends. In Afghanistan, studies found a high prevalence of depression (78.5%) and anxiety (88.9%) among women, both significantly impacting QoL.^15^ Another study reported that 65.8% of hospitalised patients had depressive symptoms, with 20.9% experiencing hypertension, both negatively affecting QoL.^16^ Similarly, in Malaysia, higher QoL among urban residents was associated with lower odds of depression and anxiety.^17^

Prior studies have demonstrated the importance of these factors for QoL. However, whether depression and anxiety mediate the link between social and biological factors and QoL is not yet known. Most existing research focuses on social and biological factors as the determinants of QoL, leaving a significant gap in understanding the mechanisms explaining the relationships between those two variables.^7 9 10 13 18^ Most existing studies focus on direct associations, without considering the mediating role of depression and anxiety in shaping QoL outcomes. Moreover, while extensive research has been conducted worldwide on the factors associated with anxiety, depression and QoL,^13 19 20^ such studies are notably limited in LMIC, specifically Indonesia. This gap is critical because the factors influencing mental health and QoL may differ in Indonesia due to varying socioeconomic conditions and the increasing burden of non-communicable diseases. Additionally, cultural differences in how mental health is perceived and addressed in the country, along with disparities in healthcare infrastructure, may result in distinct challenges that are not captured by research conducted in high-income countries. Given Indonesia’s rising burden of mental health, understanding these mediation effects is crucial for designing effective interventions. This study is the first to apply structural equation modelling (SEM) to a large population-based dataset in Indonesia to assess how depression and anxiety mediate the relationship between social and biological factors and QoL. Identifying these pathways provides critical insights for mental health policy and intervention strategies tailored to Indonesia’s unique socio-demographic and healthcare landscape.

## Objective

To examine the relationships between social and biological factors of QoL, and how depression and anxiety mediate these associations using large population-based data in Indonesia.

## Method

### Sample data

Data collection occurred from 27 July to 20 October 2023, in 12 villages across six districts/cities in four provinces on Java Island, using cross-sectional methods. Locations were purposefully selected based on urban and rural characteristics, population size and density, and the availability of primary health centres. Six villages were chosen to represent urban areas (Gunungbunder 1 in Bogor District, Cipadu in Tangerang City, Borobudur in Magelang District, Bandarhardjo and Bululor in Semarang City, and Dukuh Klopo in Jombang District), and six were chosen to represent rural areas (Gunungbunder 2 and Cibunian in Bogor District, Treko in Magelang District, Sumberaji in Jombang District, and Kasembon and Karangsari in Malang District). In each village, researchers aimed to interview 1600 individuals aged 18 and older. In Sumberaji and Treko, 341 residents from Pengampon village (Soco subvillage) were included to compensate for the shortfall in Sumberaji, where the number of residents aged 18 and over fell below 1600.

The randomisation process was conducted at the RT (neighbourhood unit) level to ensure fair participant selection. First, the number of residents in each RT was determined using population data and verified through local authorities. The number of selected RTs was calculated using the formula *1600 divided by the total population aged 18+in each village, multiplied by 100%*. RTs were then randomly chosen using STATA software to avoid selection bias, with three additional RTs selected as reserves to prevent sample shortages. All adults (18+) in the selected RTs were included as research participants. For the final RT, a random selection process ensured that every resident had an equal chance of participation.

In regard to participant consent, the procedure ensured that individuals had sufficient time to make an informed decision about their participation. Enumerators first visited potential participants’ homes, provided them with a Participant Information Sheet and allowed 24 hours for consideration. The following day, the enumerator returned to confirm their decision. If the individual agreed to participate, they were required to sign an informed consent form before the interview began, ensuring voluntary and ethical participation in the survey.

### Measures

#### Social factors

The social factor examined in this study is the individual’s social network, measured by the number of people with whom participants reported they frequently discussed important matters. This measure has been used in LMICs to identify the link between social factors and late-life depression in LMICs.^21 22^ A review, including studies from 16 LMICs, found that larger social networks, characterised by a higher number of close contacts, are indeed protective against depression among older adults.^23^ A study in Aceh, Indonesia, revealed that the number of close relationships has the most significant relationship with individuals’ mental health and overall well-being.^22^ This underscores the importance of social relationships in mental health and illustrates how the number of close ties impacts psychological well-being in LMIC settings. Unlike perceived social support, which is subjective and based on an individual’s feelings about available support, social networks offer a more objective measure by focusing on social connections’ actual number and nature. To capture this, we asked the respondents, “Are there other people with whom you often discuss things?”. If the respondent answered “yes”, we repeated this question six more times to determine the number of close social ties they identified. The final measure was obtained by summing these responses, resulting in a range from 0 to 7. For analysis, we treated social network size as a continuous variable, where higher values indicate a larger social network.

#### Biological factors

In this study, biological factors were measured by the number of comorbidities self-reported by participants. The presence of comorbidities was assessed through self-reported diagnoses on hypertension, diabetes, tuberculosis, asthma, other lung diseases, cardiovascular diseases, liver conditions, stroke, cancer and memory-related diseases. These conditions were chosen based on their inclusion in prior national health surveys in Indonesia, such as the Indonesian Family Life Survey (IFLS)^24^ and the Indonesia National Basic Health Survey (*Riset Kesehatan Dasar* or *Riskesdas*),^25^ which have consistently used similar categories to assess disease burden in the population. The category ‘other lung diseases’ was included to capture any additional chronic respiratory conditions not specifically listed, such as chronic obstructive pulmonary diseases, chronic bronchitis, emphysema or other persistent pulmonary diseases. The number ranges between 0 and 10. The variable was included as a continuous variable.

#### Quality of Life (QoL)

QoL was measured using the EuroQol Five-Dimension (EQ-5D) questionnaire, developed by the EuroQol Group in the 1980s as a concise tool for assessing health status across various disease areas.^26^ The EQ-5D has also proven effective in screening for anxiety and depressive symptoms in community settings, outperforming other self-report screening tools.^27^ It includes five dimensions: mobility, self-care, usual activities, pain/discomfort and anxiety/depression, each with three response levels: no problems, moderate problems and extreme problems. The EQ-5D score is determined by assigning a value to each dimension based on the response level and then combining these to produce an EQ-5D index score. It has been validated in Indonesia and has been used widely in health research. We used the Bahasa Indonesia version of the EQ-5D. This index score ranges from 1 (full health) to 0 (death).^26^ This study used the Great Britain value set as a reference.

### Mediating factors

#### Depression

The 10-item Centre for Epidemiologic Studies Short Depression (CES-D-10) scale was used to measure depression. This measure has been used in prior large population surveys in Indonesia.^28^ Eight items assessed negative symptoms, while two (items 5 and 8) measured positive symptoms. Participants rated the frequency of each symptom experienced ‘during the past week’ on a four-point scale. The CES-D-10 scores range from 0 to 30, with the total score calculated by summing all item scores after reversing the positive mood items. This scale has shown strong psychometric properties, including predictive accuracy and high correlations with the original 20-item version, in community populations.^29^ The Indonesia version of CESD-10 has further shown strong test-retest reliability and internal consistency, with an interclass correlation of 0.85 and a Cronbach’s α of 0.86 among individuals aged 15 and older^24^ and 0.9 among adolescents.^30^ The Cronbach’s α of the CESD-10 in this study is 0.566 (online supplemental table 1). The confirmatory factor analysis (CFA) and overall goodness-of-fit statistics of the CES-D 10 scale are available in online supplemental figure 1A and online supplemental table 2.

#### Anxiety

The seven-item Generalised Anxiety Disorder (GAD-7) scale is used to measure anxiety, which is known for its strong internal consistency and convergent validity, indicating high reliability and validity. The scale assesses symptoms such as feeling nervous, worrying, trouble relaxing and restlessness, which are common in GAD.^31^ The GAD-7 scale measures anxiety symptoms on a scale of 0 to 21. A prior study has validated GAD-7 among Indonesians and showed good internal validity and reliability with a Cronbach’s α score of 0.867 and a validity coefficient ranging from 0.648 to 0.800 (p<0.01).^32^ The reliability test of GAD-7 in this study, indicated by a Cronbach’s α, is 0.770 (online supplemental table 1). The CFA and overall goodness-of-fit statistics of the CES-D 10 scale are available in online supplemental figure 1B and online supplemental table 2.

#### Covariates

All models control for age (as continuous) and sex (male as reference), with female sex coded as 1.

### Statistical analysis

We summarised the participants’ characteristics by calculating the mean and SD for continuous variables and reporting counts and percentage for categorical variables. To compare these variables between men and women, we used the χ^2^ test for nominal and ordinal variables. For continuous variables, we reported the median and IQR. The associations between social factors, biological factors, depression, anxiety and QoL were analysed using SEM, a method widely used in epidemiological research to investigate interactions between complex variables.^33^ SEM addresses endogeneity issues and assesses direct, indirect and total effects of exogenous and endogenous factors while evaluating various hypotheses related to complex cause-and-effect interactions. We performed SEM in three models: the first modelled the association between social and biological factors and QoL; the second and third included depression and anxiety as mediators, respectively. We calculated the direct and indirect effects of social and biological factors on QoL and provided model fit statistics, including Root Mean Square Error of Approximation (RMSEA), Comparative Fit Index (CFI) and Tucker-Lewis Index (TLI), with overall goodness-of-fit statistics reported in online supplemental table 3.

For the sensitivity analysis, we included all married respondents as having their spouse as part of their social network (online supplemental figure 2A, B). We excluded the question on depression and anxiety from the EQ-5D questionnaire (online supplemental figure 2A, B). The findings are similar to the main analyses, indicating that our analyses are robust. All statistical models were estimated using STATA .17.

### Patients and public involvement

Patients and the public were actively involved in developing the questionnaire and the pilot testing phase of this study. During the questionnaire design process, we sought input from individuals representing the target population to ensure that the survey items were culturally appropriate, clearly understood and relevant to the participants with lived experiences. A pilot study was conducted before the main data collection to test feasibility, refine wording and improve clarity based on feedback from community members and stakeholders. Their insights helped enhance the comprehensibility and applicability of the measures used in this study.

## Findings

The study included a total of 19 236 participants, of whom 849 (4.42%) and 2339 (12.17%) were identified as depressed using CESD-10 and anxious using GAD-7. Table 1 details the demographic characteristics of the sample, categorised by sex: 10 586 participants were female and 8643 were male. The median age of female participants was 42 years old (IQR=24), slightly younger than male participants (median 43, IQR=25). The majority of female participants were married (73.52%) and had attained junior high school education (45.93%) or primary education and lower (47.35%). More than half of women were housewives (56.87%), while men were predominantly engaged in casual work (35.57%). Regarding social networks, most participants reported having only one close person, with 69.68% of women and 66.23% of men falling into this category. The median and IQR of the CESD-10 scores are similar among men and women (median=2, IQR=4).

**Table 1:** Descriptive statistics on individual characteristics

No.	Variables	Femalen=10 586		Malen=8643		P value	Missing data
		n/median	%/IQR	n/median	%/IQR		N (%)
1	Age (years), median (IQR)	42	24	43	25	0.545	7 (0.04)
2	Education					<0.001	12 (0.06)
	Primary and lower	5012	47.35	3555	41.13		
	Junior high school	4862	45.93	4617	53.42		
	Senior high school and higher	705	6.66	466	5.39		
3	Marital status					<0.001	7 (0.04)
	Married	7783	73.52	6451	74.64		
	Widowed	1449	13.69	361	4.18		
	Divorced	277	2.62	135	1.56		
	Single	1074	10.15	1692	19.58		
4	Employment status					<0.001	5 (0.03)
	Self-employed	1297	12.25	1676	19.39		
	Government employee	90	0.85	90	1.04		
	Private employee	681	6.43	1217	14.08		
	Casual worker	944	8.92	3074	35.57		
	Unemployed	758	7.16	1135	13.13		
	Retired	44	0.42	105	1.21		
	Housewife	6020	56.87	91	1.05		
	Student	279	2.64	263	3.04		
	Others	472	4.46	988	11.43		
5	Ever hypertension	1373	12.97	561	6.49	<0.001	0
6	Ever diabetes	402	3.80	214	2.48	<0.001	0
7	Ever tuberculosis	45	0.56	48	0.43	<0.001	1 (1.06)
8	Ever asthma	176	1.66	147	1.70	0.922	0
9	Ever other lung diseases	114	1.08	135	1.56	<0.001	1 (0.40)
10	Ever heart attack, coronary heart disease or other heart problems	145	1.37	96	1.11	0.263	0
11	Ever liver disease	80	0.76	102	1.18	0.010	0
12	Ever stroke	67	0.63	88	1.02	0.012	0
13	Ever cancer	34	0.32	9	0.10	0.007	0
14	Ever memory-related disease	25	0.24	15	0.17	0.633	0
15	Number of comorbidities, median (IQR)	0	0	0	0	<0.001	0
16	Social network, median (IQR)	1	0	1	0	<0.001	0
17	Depression, median (IQR)	2	4	2	4	0.036	28 (0.15)
18	Anxiety, median (SD)	1	2	1	3	<0.001	17 (0.09)

**Figure 1:**
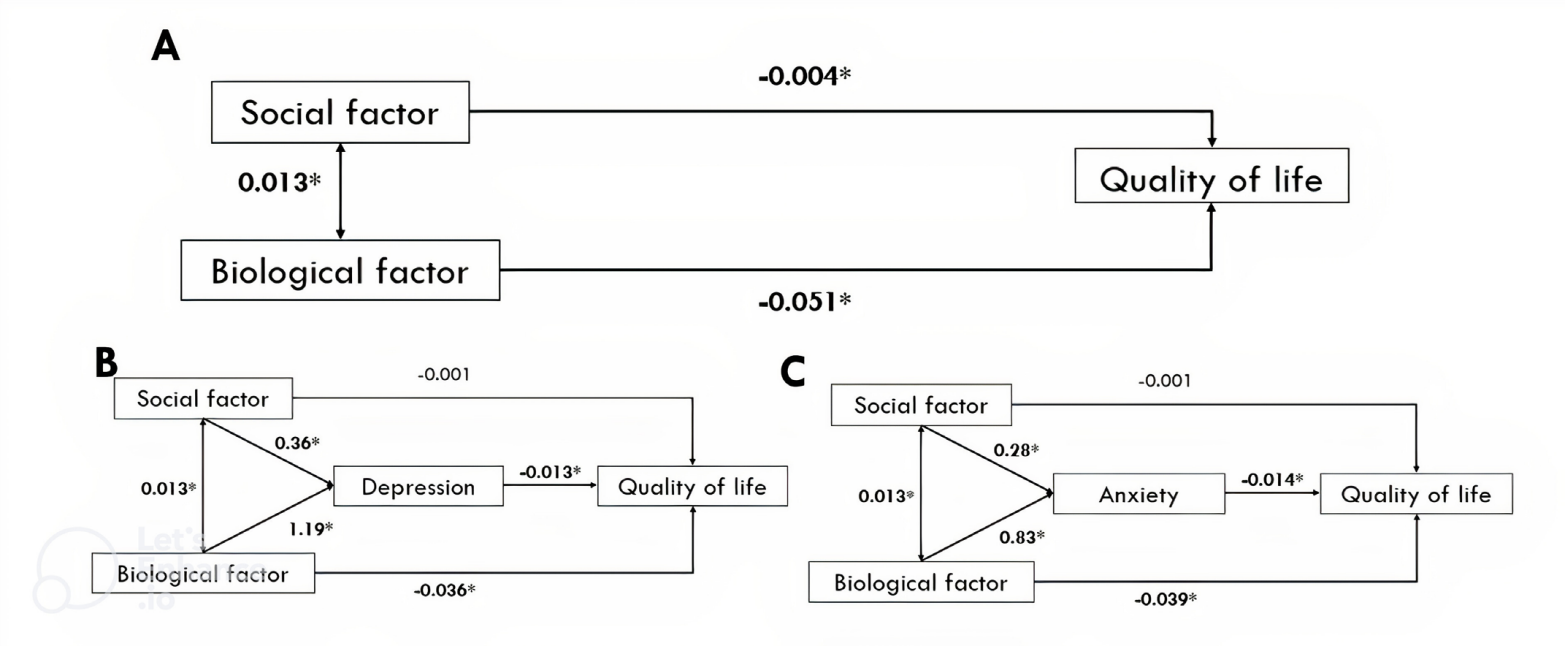
Structural equation models to identify (A) the association between social factors, biological factors and quality of life (QoL); (B) depression mediates the relationship between social factors, biological factors and QoL; and (C) anxiety mediates the relationship between social factors, biological factors and QoL.

In this study, we performed three models, each incorporating all the covariates. In the first model (figure 1A), after controlling for age and sex, social factors (β=−0.004, p<0.001) and biological factors (β=−0.05, p<0.001) were associated with QoL. In the second model (figure 1B), we found that depression mediated the relationship between social and biological factors of QoL. Social factors were associated with biological factors in all models (β=0.013, p<0.001).

In the second model (figure 1B), the analysis revealed that the direct relationship between social factors and QoL was not significant, implying that the link between social factors and QoL is fully mediated by depression (β=−0.001, p=0.588). Social factors were significantly associated with depression (β=0.36, p<0.001). The direct effect of biological factors on QoL remained significant (β=−0.036, p<0.001) in the second model. Biological factors were significantly associated with depression (β=1.19, p<0.001). Higher depression scores were associated with lower QoL (β=−0.013, p<0.001).

In the third model (figure 1C), where the mediator was anxiety, the results were similar to the second model. Social factors were significantly associated with anxiety (β=0.28, p<0.001), but not directly associated with QoL (β=−0.001, p=0.66). Biological factors were significantly associated with both anxiety (β=0.83, p<0.001) and negatively impacted QoL (β=−0.039, p<0.001). This indicates that biological factors impact QoL both directly and indirectly through depression or anxiety, suggesting that comorbidities were associated with QoL via both pathways. Additionally, the indirect path from social or biological factors to QoL, mediated by anxiety, was significant and negative (β=−0.014, p<0.001). All three models showed excellent fit, with similar values across the indices, as detailed in online supplemental table 3.

Table 2 presents the magnitude of the direct, indirect and total effects of the models. The direct effect is the unmediated effect of social or biological factors on QoL. The indirect effect is the effect which passes through the mediator, and the total effect is the combination of the two. In the second model, 29.30% of the effect of biological factors was mediated by depression. Anxiety mediated 22.83% of the effect of biological factors on QoL. Both depression and anxiety fully mediated the links between social factors and QoL. Model fit analysis is provided in online supplemental table 3.

**Table 2:** Total and direct effects of each variable on quality of life

Model	Main predictors	Mediator	Total effect	Direct effect	Indirect effect	Proportion mediated
2	Social factor	Depression	−0.005**	−0.001	−0.004***	84.39%
	Biological factor	Depression	−0.050***	−0.036***	−0.014***	29.30%
3	Social factor	Anxiety	−0.005***	−0.001	−0.004***	87.17%
	Biological factor	Anxiety	−0.049***	−0.039***	−0.010***	22.83%

*p<0.05, **p<0.005, ***p<0.001.

## Discussion

This study provides the first evidence from a large population cohort representative for 12 districts in four provinces in Indonesia that depression and anxiety mediate the relationship between biological and social factors and QoL. Specifically, the direct relationship between biological factors and QoL was reduced by almost one-third when accounting for the mediation effects of depression and anxiety. Biological factors consistently demonstrated a stronger direct association with depression, anxiety and QoL compared with social factors in all models. Additionally, while the direct relationship between biological factors and QoL remained significant, the relationship between social factors and QoL became insignificant after accounting for depression and anxiety as mediators. This indicates that the link between social factors and QoL is largely indirect, channelled through the mediation of depression and anxiety.

We identified a significant association between an increased number of comorbidities and a lower QoL, consistent with a study using the Dutch National Health Survey, which demonstrated a noticeable decline in QoL as comorbidities increase.^19^ The study highlighted that individuals with diabetes and comorbid conditions experience significantly lower scores in both mental well-being and physical function. A hospital-based cross-sectional study in Bangladesh also confirmed this inverse relationship, particularly evident among individuals with hypertension.^34^ Notably, diabetes and obesity emerged as the most prevalent comorbidities associated with the highest EQ-5D mean utilities.

One possible mechanism by which a greater number of comorbidities related to lower QoL is highlighted in a recent study using data from the IFLS, particularly in cases involving non-communicable diseases (NCDs). The study found that individuals with three or more NCDs experienced significant productivity loss, with only 49.8% employed compared with 84.3% without NCDs. Additionally, people with multiple NCDs reported missing more days of primary daily activities and spending more days confined to bed, ultimately lowering QoL.^35^ Alternatively, a study using WHO’s Study on Global AGEing and Adult Health data found that adults with three or more chronic diseases experienced substantially higher levels of depression compared with those with one or two chronic diseases, indicating that managing multiple health conditions significantly contributes to mental health deterioration.^36^ This is particularly concerning in LMICs like Indonesia, where the burden of NCDs is rapidly increasing. In line with this, the IFLS study found that individuals with multiple NCDs had significantly higher rates of outpatient and inpatient visits, and catastrophic health expenditures, further worsening their reduced QoL.^35^

This study found that the connection between biological factors and QoL is partly mediated by anxiety and depression. Specifically, having more comorbidities was associated with increased levels of anxiety and depression,^37^ suggesting that the emotional and physical burden of managing multiple chronic conditions, including changes in lifestyle, contributes to increased psychological distress. Moreover, higher levels of comorbidities were linked to lower QoL,^34^ indicating that multiple health conditions negatively impact overall well-being through heightened levels of anxiety and depression. These findings are consistent with a biopsychosocial model,^38 39^ which suggests that biological, psychological and social factors influence QOL. Adopting this model, a study among multiple sclerosis patients found that beyond the physical symptoms, psychological factors such as depression and anxiety, along with social elements like support systems, play crucial roles in influencing QoL.^39^ Another study used the biopsychosocial model to demonstrate that depression mediated the relationship between baseline pain catastrophising and the change in mental QoL after a year, highlighting the importance of psychological distress in explaining variations in well-being among patients with long-term illnesses.^40^ Future research should focus on developing integrated, personalised and scalable interventions that address both physical and mental health needs, as the psychological strain of managing various conditions can create a cycle where declining mental health further harms physical health and QoL.

We found that a higher number of close people is significantly linked to lower QoL. This discovery contrasts with a previous study among older adults in Central and Eastern Europe, which reported that a higher frequency of contact was associated with increased QoL for every unit increase.^20^ Additionally, research from a nationally representative US cohort revealed that stronger friendships improved various health and psychosocial indicators, including reduced mortality risk, increased physical activity, higher levels of positive affect and mastery, and lower levels of negative affect and depression, although friendships were also associated with a higher likelihood of smoking and, to a lesser extent, heavy drinking, which did not reach conventional levels of statistical significance.^41^

The potential explanation for how having more friends can reduce QoL is detailed in research conducted by Glover and Parry.^42^ Their study identified situations where friendships imposed burdens on participants. These burdens primarily stemmed from social norms and expectations within these friendships, which pressured individuals to participate in activities they found stressful. These norms included obligations to attend social events, offer emotional support or conform to group behaviours that did not necessarily align with the participants’ preferences or comfort levels. As a result, these perceived burdens could elevate stress and discomfort levels, ultimately impacting individuals’ overall QoL.

Moreover, the current study revealed that the presence of depression and anxiety mediates the association between social factors and QoL. As levels of depression and anxiety increase, they effectively explain and account for how social factors are related to QoL outcomes. Furthermore, the study found that higher levels of depression and anxiety correlate with lower overall QoL scores. This suggests that increasing levels of depression and anxiety are related to lower QoL among the study participants.

The addition of depression and anxiety as mediating factors eliminated the direct link between social factors and QoL, while the direct association between biological factors and QoL remained unaffected. This suggests that any association between social factors and QoL is entirely channelled through depression and anxiety. Prior studies in LMICs revealed the relationships between depression and QoL.^43 44^ For example, Mohammadi *et al* found that depression negatively affected both the physical (adjusted odds ratio (aOR) =0.966, p=0.0186) and psychological (aOR=0.950, p=0.0005) domains of QoL among healthcare workers in Herat province, Afghanistan.^43^

This study exhibits several key strengths. First, it includes a large population cohort of over 19 000 participants, providing robust statistical power and generalisability in urban and rural communities in the four provinces in Java, Indonesia. Moreover, comprehensive measures for both depression and anxiety allow for a thorough examination of their respective roles and interactions in influencing QoL outcomes, contributing significantly to the depth and validity of the study’s findings.

This study presents several limitations. The cross-sectional design prevents us from establishing causal relationships between variables, as it captures data at a single point in time. Future research should implement longitudinal designs to understand better the temporal sequencing and directionality of the relationships between biological and social factors, mental health and QoL. To address this limitation, we plan to collect a second wave of data in 2025, allowing for a longitudinal follow-up analysis, which will be the first longitudinal study focusing on mental health, risk factors and healthcare utilisation in Indonesia. This will enable us to assess changes in depression, anxiety and QoL over time, providing stronger evidence for mediation effects and long-term intervention implications. Additionally, the information on comorbidities was self-reported, which could introduce biases such as recall inaccuracies or social desirability effects, potentially compromising the accuracy of the findings.^45^ Similarly, self-reported health conditions might be influenced by underdiagnosis or selective reporting. Therefore, caution is advised when interpreting the results concerning the biological factors, and future studies should aim to incorporate more objective measures to strengthen the robustness of their conclusions. Another limitation is that we employed a structured randomisation process at the RT (neighbourhood unit) level, ensuring fair selection within each village. Although this method minimised selection bias, the population distribution between urban and rural settings was not strictly proportionate. Future studies should consider proportionate weighting or stratified random sampling to enhance demographic representativeness. Finally, this study has a relatively low internal consistency of the CES-D-10 scale (Cronbach’s alpha=0.566). While this value is lower than the conventional threshold for strong reliability, it is still within an acceptable range for short screening tools, particularly in diverse population-based studies. The CES-D-10 has been widely used in mental health research, including in Indonesia, and remains a practical and efficient tool for detecting depressive symptoms in large-scale surveys. Although a lower Cronbach’s alpha may indicate some variability in responses, our findings remain robust as they align with existing research on depression and quality of life.

## Conclusion

This study found that depression and anxiety significantly mediate the relationship between social and biological factors and QoL in Indonesia. To improve mental health and QoL in Indonesia, proactive promotion and prevention strategies must be implemented at individual and community levels. Public health initiatives should focus on enhancing physical and social well-being by promoting healthy lifestyles, encouraging regular physical activity and improving nutrition through government-supported wellness programmes. Additionally, strengthening social networks is crucial, particularly in rural and low-resource settings where access to mental healthcare is limited. Social isolation is a known risk factor for poor mental health outcomes, and interventions aimed at enhancing social connections have been shown to improve well-being, engagement in care and quality of life among individuals with mental health conditions.^46^ Community-based interventions, such as leveraging Posyandu (integrated health posts) to deliver psychoeducation, stress management workshops and peer support groups, can foster social connections and reduce the stigma around mental health.

Given that depression and anxiety were only screened in this study, early intervention programmes—such as scaling up mental health screening at Puskesmas (community health centres) and integrating psychological support into Indonesia’s National Health Insurance Scheme (Badan Penyelenggara Jaminan Sosial or BPJS)—could prevent worsening conditions. Expanding digital mental health platforms, including SMS-based counselling and telepsychiatry services, can help reach underserved populations. Integrating these strategies within primary care, schools and workplaces ensures a holistic and sustainable approach to improving mental health and QoL in Indonesia.

## Supplementary material

10.1136/bmjopen-2024-095110 online supplemental file 1

## Data Availability

Data are available upon reasonable request.
