# Supplementary material for: Exploring the mediating role of depression and anxiety in the relationship between social and biological factors and quality of life in Indonesia: a structural equation modelling approach
Source: BMJ Open. 2025 Apr 28;15(4):e095110. doi: 10.1136/bmjopen-2024-095110 (PMC12039030; doi:10.1136/bmjopen-2024-095110)
Supplement: online supplemental file 1 [file bmjopen-15-4-s001.docx]

# SUPPLEMENTARY

## Validity & Reliability Test

The data collection utilised measurements such as GAD-7 and CES-D. A cross-sectional study by Idaiani et al. [1] involving 1,020 subjects found that the inter-item correlation and item-rest correlation of the 7-item GAD-7 questionnaire ranged from 0.57 to 0.83, with all p-values less than 0.050, and a Cronbach's alpha of 0.88, indicating high reliability for measuring anxiety in the non-healthcare worker population. Another study compared GAD-7 results with the Mini International Neuropsychiatric Interview International Classification of Diseases-10 (MINI ICD-10) as the gold standard [2]. This study found that the content validity coefficient of GAD-7 was 0.847. The internal validity test using Spearman correlation showed a coefficient ranging from 0.648 to 0.800, with all p-values less than 0.050. The sensitivity and specificity of the GAD-7 were 100% and 84.4%, respectively, with a cut-off point of ≥7. These findings demonstrate that the Indonesian version of the GAD-7 is valid, reliable, and accurate as a screening tool for Generalized Anxiety Disorder (GAD) in adults.

In regards to the CES-D scale, an analysis using data from the fifth wave of the Indonesia Family Life Survey (IFLS) indicated generally favourable psychometric properties, such as item discrimination, location, and fit, with the exception of items 5 and 8 which exhibited poorer quality [3]. Despite these shortcomings, the overall reliability coefficient, inclusive of all items, met established standards for reliability in measurement. Moreover, the scale proved to be rich in its ability to assess moderate to severe levels of depressive symptoms, providing valuable information in this regard.

**Supplementary Table 1.** Reliability Analysis of Psychological Scales: CESD and GAD-7

| **Scale** | **Items** | **Cronbach’s alpha** |
| --- | --- | --- |
| CESD | 10 | 0.566 |
| GAD-7 | 7 | 0.770 |

Supplementary Table 1 presents the reliability analysis of psychological scales using Cronbach's alpha, which measures internal consistency. According to the table, the CESD scale has a low Cronbach’s alpha (0.566), indicating questionable reliability. This suggests that the items in the CESD scale may not be consistently measuring the same underlying construct of depression. While the GAD-7 scale has a Cronbach’s alpha of 0.770, indicating acceptable reliability. This implies that the items in the GAD-7 scale are fairly consistent in measuring the construct of generalised anxiety.


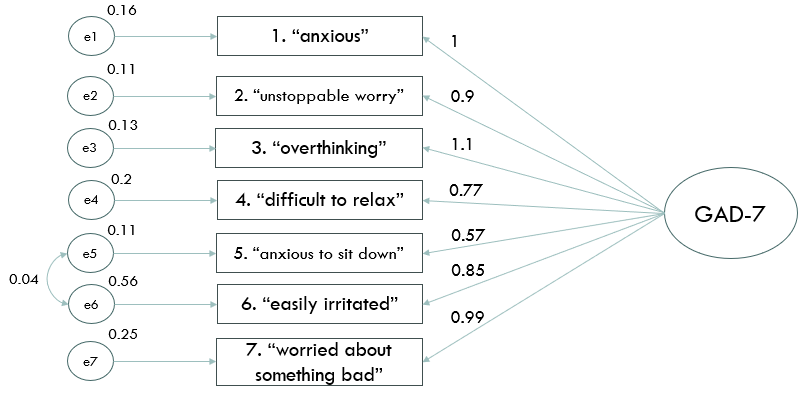
**Supplementary Figure 1**. Confirmatory Factor Analysis (CFA) Model for the CES-D and GAD-7 Scales

**B**


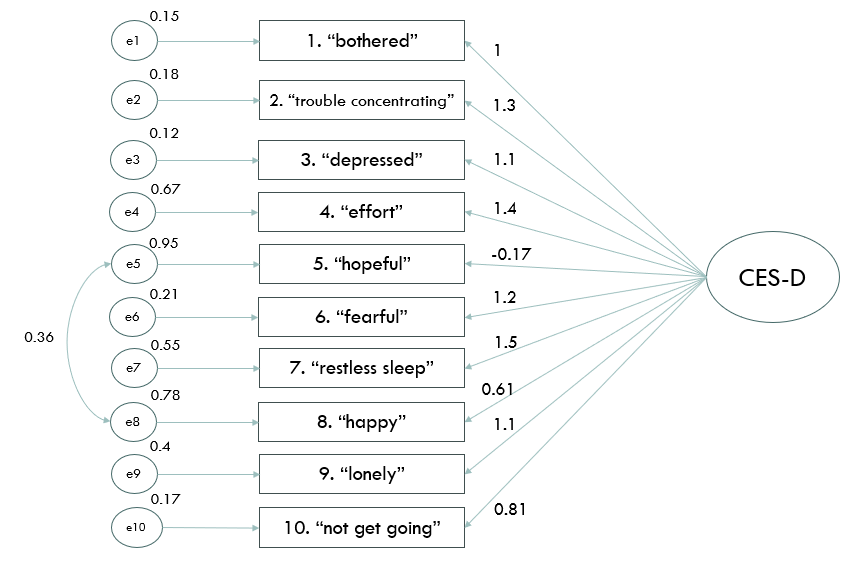


**A**

**Supplementary Figure 1A** represents a Confirmatory Factor Analysis (CFA) model for the CES-D, aimed at improving model fit by correlating error terms, specifically q113 (e5) and e8 (q116), which correspond to the item’s "effort" and "happy." Each observed variable (e.g., "bothered," "trouble concentrating") is linked to the latent variable (CES-D), with factor loadings indicating the strength of these relationships. Notably, the items "restless sleep" and "effort" had the highest factor loadings, with values of 1.5 and 1.4, respectively, indicating strong correlations with the underlying construct of depression. The CFA model, enhanced by the added correlation between the error terms for "effort" and "happy," suggests an improved fit, acknowledging the potential shared variance between these items and thus increasing the model's accuracy in measuring depression. This adjustment underscores the importance of considering item interrelationships and the underlying factors that may influence responses, ultimately providing a more reliable measurement model for the CES-D scale.

Furthermore, **Supplementary Figure 1B** presents a CFA model for the GAD-7, with an attempt to improve model fit by correlating error terms specifically for items e6 (q107) "easily irritated" and e7 (q108) "worried about something bad". In this model, "overthinking" and "anxious" exhibited the highest factor loadings, with values of 1.1 and 1, respectively, indicating strong correlations with the underlying construct of generalised anxiety. The addition of the correlation between the error terms for "easily irritated" and "worried about something bad" suggests an improved model fit, recognising potential shared variance between these items and thus enhancing the overall accuracy of the model in measuring generalised anxiety.

**Supplementary Table 2**. Overall goodness-of-fit statistics of each measurement scales

| Scales | Fit Indices | | | |
| --- | --- | --- | --- | --- |
|  | SRMR | RMSEA | CFI | NNFI (TLI) |
| CES-D | 0.023 | 0.036 | 0.966 | 0.956 |
| GAD-7 | 0.023 | 0.059 | 0.974 | 0.959 |

**Supplementary Table 2** summarises the fit indices for all measurement scales, including SRMR, RMSEA, CFI, and NNFI or TLI. According to the guidelines for SEM [4], both the CES-D and GAD-7 scales demonstrated good fit, with similar values across the indices. Specifically, the SRMR values for these scales were less than 0.05, indicating a very good fit. The RMSEA values were also less than 0.05, which indicates a close fit. Additionally, both the CFI and TLI scores for CES-D and GAD-7 were above 0.95, further indicating a very good fit.

**Supplementary Table 3**. Overall goodness-of-fit statistics of each model

| Fit Index | Model | | |
| --- | --- | --- | --- |
|  | 1A | 1B | 1C |
| SRMR | <0.001 | <0.001 | <0.001 |
| RMSEA | <0.001 | <0.001 | <0.001 |
| CFI | 1.000 | 1.000 | 1.000 |
| NNFI (TLI) | 1.000 | 1.000 | 1.000 |

**Supplementary Table 3** summarises the fit indices for all models, including Standardised Root Mean Square Residual (SRMR), Root Mean Square Error of Approximation (RMSEA), Comparative Fit Index (CFI), and Non-Normed Fit Index (NNFI or TLI). According to the guidance for structural equation modelling [4], all three models demonstrated excellent fit with similar values across the indices. Specifically, the SRMR values were less than 0.001, indicating a small discrepancy between the observed and predicted correlations. The RMSEA values were also less than 0.001, suggesting a close fit of the model in relation to the degrees of freedom. Additionally, both the CFI and the TLI had perfect scores of 1.00, reflecting an ideal fit for the data. These fit indices collectively indicate that all three models fit the data exceptionally well, demonstrating robustness and reliability in their structural equation modelling.

**Supplementary Figure 2**. Modified structural equation models. Note: *p <0.001.


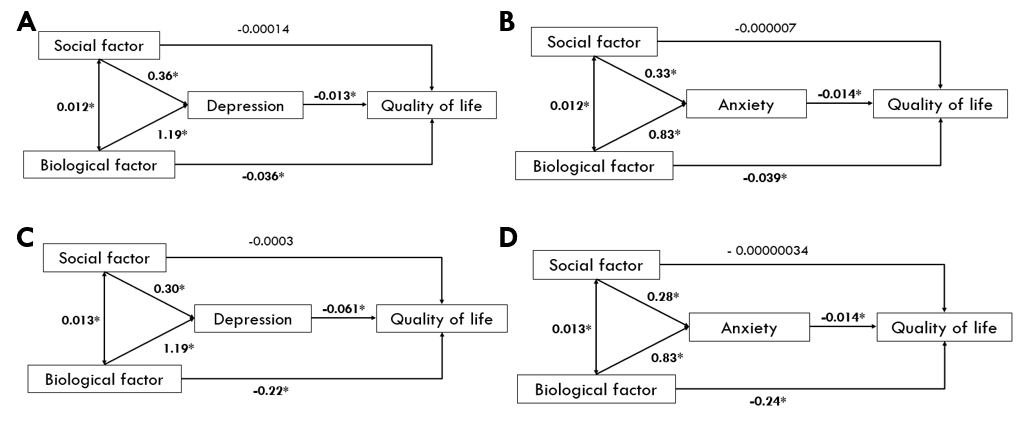


For the sensitivity analysis, we included all married respondents as having their spouse as part of their social network (**Supplementary Figures 2A and 2B**) and excluded the question on depression and anxiety from the EQ-5D questionnaire (**Supplementary Figures 2A and 2B**). When we modified the social factor by adding one point to the scores of married individuals, even if they reported having no close friends, the direct effect on QoL remained insignificant, mirroring the results of the original model, indicating that our analyses are robust.

## Supplementary References

1. Idaiani S, Herawati MH, Mubasyiroh R, Indrawati L, Yunita I, Sitorus N, et al. Reliability of the General Anxiety Disorder -7 Questionnaire for Non-Healthcare Workers The 8th International Conference on Public Health 2021. doi:10.3889/oamjms.2022.9999.

2. Larasari A, Khamelia, Budikayanti A, Prihartono J. Uji Validitas, Uji Reliabilitas dan Uji Diagnostik Instrumen Generalized Anxiety Disorder-7 (Gad-7) Versi Bahasa Indonesia Pada Pasien Epilepsi Dewasa [online]. 2015.<https://lib.ui.ac.id/detail?id=20424638&lokasi=lokal>. (*Validity, Reliability, And Diagnostic Tests Of Generalized Anxiety Disorder-7 (Gad-7) Instrument – Indonesian Version In Adult Epilepsy Patients*)

3. Marvianto RD. Psychometric properties of Mental Health Scale: An Item Response Theory Approach. Gadjah Mada Journal of Psychology (GamaJoP) 2023;9(2). doi:10.22146/gamajop.73878.

4. Hooper D, Coughlan J, Mullen MR. Structural Equation Modelling: Guidelines for Determining Model Fit Daire Hooper1 [online]. 2008. https://academic-publishing.org/index.php/ejbrm/article/view/1224 (accessed: 26 June 2024).
