# Supplementary material for: Exploring the mediating role of depression and anxiety in the relationship between social and biological factors and quality of life in Indonesia: a structural equation modelling approach
Source: BMJ Open. 2025 Apr 28;15(4):e095110. doi: 10.1136/bmjopen-2024-095110 (PMC12039030; doi:10.1136/bmjopen-2024-095110)
Supplement: Supplementary file 2 [file bmjopen-2024-095110.reviewer_comments.pdf]

PEER REVIEW HISTORY

BMJ Open publishes all reviews undertaken for accepted manuscripts. Reviewers are asked to complete a checklist review form and are provided with free text boxes to elaborate on their assessment. These free text comments are reproduced below.

ARTICLE DETAILS

Title (Provisional)

Exploring the mediating role of depression and anxiety in the relationship between social and biological factors and quality of life in Indonesia: A structural equation modelling approach

Authors

Susanti , Herni; Fajri, Mashita; Keliat, Budi Anna; Brooks, Helen; Bee, Penny; Maharani, Asri

---

VERSION 1 - REVIEW

**Reviewer**	**1**
**Name**	**Neyazi, Ahmad**
**Affiliation**	**Afghanistan Center for Epidemiological Studies**
**Date**	**21-Dec-2024**
**COI**	**None**

This study "Depression and anxiety mediating the link between social and biological factors and quality of life in Indonesia: A structural equation modelling" investigated how social and biological factors influence quality of life (QoL) and the mediating roles of depression and anxiety using data from a large household survey in Indonesia. The cross-sectional analysis included 19,236 adults (mean age 43.23, 55% female). Depression, anxiety, and QoL were assessed using validated scales (CESD-10, GAD-7, and EQ-5D, respectively). Social factors were measured by the number of close personal relationships, and biological factors were assessed by self-reported physical comorbidities. Structural Equation Modelling (SEM) revealed that both social (β= -0.004, p<0.001) and biological factors (β= -0.051, p<0.001) were associated with QoL. The effect of biological factors on QoL was partially mediated by depression and anxiety, while the effect of social factors was fully mediated by these mental health conditions. These findings highlight the importance of addressing depression and anxiety in individuals with comorbidities to mitigate QoL decline.

Here are my comments:

Abstract

1. While the abstract summarizes the study, consider simplifying the language to make it more accessible to a broader audience.

2. Include more specific statistical results (e.g., effect sizes or percentages) to provide a clearer understanding of the findings.

3. Strengthen the conclusion by emphasizing the broader implications for research or policy.

Introduction

1. While comprehensive, some statements could be supported with more recent or region-specific references to better contextualize the study (e.g., DOI: 10.1136/bmjopen-2023-071939) (DOI: 10.1038/s41371-024-00914-5).

2. Clearly delineate the novelty of examining mediation effects of depression and anxiety and quality of life in the Indonesian context.

3. Ensure that the stated objectives align with the methodology and findings detailed later.

Methods

1. Provide more detail about the randomization process and any potential biases in participant selection.

2. Highlight the limitations of the CES-D scale's low Cronbach's alpha (0.566) and discuss its impact on the reliability of depression measurements.

3. While ethical approvals are noted, consider expanding on participant consent procedures to ensure clarity.

Discussion

1. Expand the discussion to compare findings with a broader range of international studies (e.g., 10.1186/s40359-023-01059-9).

2. Elaborate on the theoretical pathways through which depression and anxiety mediate the effects of social and biological factors on QoL (e.g., DOI: DOI: 10.1093/pubmed/fdae111).

3. Discuss potential biases, including self-reporting and the cross-sectional design, in greater detail.

4. Emphasize specific recommendations for interventions in LMICs, tailored to the Indonesian context.

Conclusion

1. Condense the conclusion to focus on the most critical findings and their implications.

2. Provide a more detailed outline of proposed longitudinal studies or interventions to address the identified research gaps.

Reviewer 2

**Name**	**Ogundare, Temitope**
**Affiliation**	**Boston University**
**Date**	**20-Jan-2025**
**COI**	**None**

Reviewer's comment— Depression and anxiety mediating the link between social and biological factors and quality of life in Indonesia: A structural equation modelling

1. Abstract
    a. The methods section—describe the methods only, results (including number of participants and mean age) should not be included.
2. Introduction
    a. Page 3, line 53 – 55: the authors should elaborate more on the connection between social and biological factors and QoL—with reference to extant literature and what the gaps in the literature are. As it stands there is no clear rationale for the study.
    b. The authors mentioned social and biological factors influencing quality of life but did not fully explore this. What are these social and biological factors? Has existing literature found similar associations between these factors and anxiety and depression? any existing theories or works that have suggested that anxiety and depression may mediate the relationship between social and biological factors and quality of life?
3. Methods
    a. Sample data:
        i. The authors sampled 12 villages – 6 rural and 6 urban. It is not clear how these 12 villages were chosen. How did the author avoid selection bias?
        ii. Given the difference in population between rural and urban villages, a proportionate sampling technique should have been employed. This will ensure that the sample will be representative of the population.
    b. Measures
        i. Social factors: why did the author choose social network as the sole social factor to identify. Is this based on literature review?
        ii. Also, there are standardized measures that have been validated for measuring social network; why did the authors not use those?
        iii. Social network is a complex construct, it is unlikely that one question would measure the construct. Therefore, the validity of the construct is questionable.
        iv. Also, it is unclear how a Likert scale with 8 responses was generated for the social network question. The phrasing is most suitable for a yes or no answer. The validity of the construct is questionable.
        v. Biological factors: recall bias. What about access to care—if these conditions are present but not diagnosed. Also, it is not clear what the authors mean by "the number ranges between 0 and 10". Does this mean that each condition is given a score of 1? Also, there are a lot of

chronic conditions, how did the authors settle on the ones included in the survey? Also, ‘other lung diseases’ could mean a host of conditions from acute to chronic lung diseases—were the authors specific on the chronicity?

   vi. Quality of life measure: why did the author use the Great Britian value? Also, was this instrument translated into the local language?

c. Mediating factors
   i. CES-D-10: ‘The reliability test, with a Cronbach's alpha of 0.566, and the model fit for CFA are detailed in Supplementary 1 and 2A.”—did the authors conduct a validation study on the CES-D? If yes, was this part of the current study? did they use the same sample as for the main study?
   ii. Similar question for the GAD-7: the reported Cronbach’s alpha, was this part of the current study and using the same sample?

d. Statistical analysis
   i. “We summarised the participants’ characteristics by calculating the mean and standard deviation (SD) for continuous variables and reporting counts and percentage for categorical variables. To compare these variables between males and females, we utilised the chi-square test for nominal and ordinal variables and the Kruskal-Wallis test for continuous variables”— The Kruskal-Wallis test assumes that the data are non-parametric, meaning they do not follow a normal distribution. Therefore, it would be more appropriate to report the median and interquartile range rather than the mean and standard deviation as measures of central tendency and variability, as the latter may not accurately represent the data distribution.

4. Discussion\
   a. Page 9, line 34 – 50. The authors discuss how comorbidities impact quality of life by citing factors such as loss of productivity, increased healthcare expenditure, and more frequent hospital visits. While loss of productivity and increased healthcare spending can be considered social factors that may mediate the relationship between comorbidities and quality of life, this study focuses solely on social networks. As a result, the model may not fully capture the broader influence of social determinants on quality of life, potentially limiting the generalizability of the findings
   b. Page 9, lines 53- 57: “Specifically, having more comorbidities was associated with increased levels of anxiety and depression, suggesting that the emotional and physical burden of managing multiple chronic conditions, including changes in lifestyle, contributes to increased psychological distress”—citation needed
   c. Page 10, lines 29 – 36: The authors assert that depression and anxiety fully mediate the relationship between social factors and QoL. While their model suggests full mediation, the interpretation should acknowledge that statistical mediation does not imply causality. The cross-sectional nature of the study limits the ability to establish directionality, and unmeasured confounders or alternative pathways may still exist. Additionally, the statement that

"increasing levels of depression and anxiety lead to a reduction in QoL" implies a causal effect, which is not supported by mediation analysis alone. A more cautious interpretation should emphasize that depression and anxiety statistically account for the relationship rather than determining it exclusively. The authors should also clarify whether model fit and potential confounding variables were adequately addressed to support the claim of full mediation

   d. Page 10, line 38 – 49: The statement overstates the findings by implying that social factors impact QoL entirely through depression and anxiety, especially considering that only one social factor—social network—was examined in the study. Mediation analysis demonstrates statistical relationships but does not establish causality or preclude other direct or indirect pathways. The authors should adopt more cautious language to reflect the observational nature of the study. Furthermore, while the discussion of biological mechanisms is relevant, its connection to QoL remains unclear, as it does not explicitly explain how inflammation directly contributes to reduced quality of life

5. Study strengths
   a. Page 10, lines 50 -60: The study's large sample size enhances statistical power, but generalizability claims should be made cautiously. The selection of 12 villages—6 urban and 6 rural—without clear selection criteria raises concerns about representativeness. The reported "randomised sampling method" requires clarification, as the equal allocation of participants may not reflect actual population distributions as rural and urban populations differ in size. Proportionate sampling would improve representativeness and reduce bias.The claim of providing a "nuanced understanding" should be framed more cautiously to reflect the study's observational nature.
6. Supplementary materials
   a. It remains unclear whether the CFA results shown are for the present study or reported from another study. If from present study, was this a pilot or part of the main study?
   b. If previous validation studies on the GAD-7 and CES-D have been conducted, they should be included in the main manuscript as part of the survey instrument description, rather than in the supplementary section.

VERSION 1 - AUTHOR RESPONSE

Comments from Reviewer #1

Abstract

1. While the abstract summarizes the study, consider simplifying the language to make it more accessible to a broader audience.

Authors' response

Thank you for the comment. We have simplified the language used in the Abstract (page 1) for broader audience.

Comments from Reviewer #1

2. Include more specific statistical results (e.g., effect sizes or percentages) to provide a clearer understanding of the findings

Authors' response

We have revised the Finding section in the Abstract (page 1) into:

"*Findings: Among participants, 849 (4.42%) had depression and 2,339 (12.17%) had anxiety. SEM analysis showed that social factors (ß=-0.004, p<0.001) and biological factors (ß=-0.051, p<0.001) were directly associated with QoL. Mediation analysis revealed that depression and anxiety fully mediated the relationship between social factors and QoL. For biological factors, depression and anxiety partially mediated the relationship with QoL, accounting for 29.30% and 22.83% of the total effect, respectively."*

Comments from Reviewer #1

3. Strengthen the conclusion by emphasizing the broader implications for research or policy

Authors' response

We have added the broader implications of our findings for research and policy in the Abstract (page 1):

"*Depression and anxiety mediate the relationship between social and biological factors and QoL. Strengthening social support and improving mental health access can mitigate these risks. Future research should examine long-term trends and intervention effectiveness to inform targeted policies for at-risk populations."*

Comments from Reviewer #1

Introduction

4. While comprehensive, some statements could be supported with more recent or region-specific references to better contextualize the study (e.g., DOI: 10.1136/bmjopen-2023-071939) (DOI: 10.1038/s41371-024-00914-5)

Authors' response

Thank you for the suggestion. We have included recent studies in LMICs and Southeast Asia region in the Introduction section (page 3) to contextualise the study better:

"*Research in LMICs shows similar trends. In Afghanistan, studies found a high prevalence of depression (78.5%) and anxiety (88.9%) among women, both significantly impacting QoL [15]. Another study reported that 65.8% of hospitalised patients had depressive symptoms, with 20.9% experiencing hypertension, both negatively affecting QoL [16]. Similarly, in Malaysia, higher QoL among urban residents was associated with lower odds of depression and anxiety [17]."*

Comments from Reviewer #1

5 Clearly delineate the novelty of examining mediation effects of depression and anxiety and quality of life in the Indonesian context

Authors' response

We have highlighted the novelty of exploring how depression and anxiety mediate the relationship between social and biological factors and quality of life in the Indonesian context (page 3 – 4):

"Most existing studies focus on direct associations, without considering the mediating role of depression and anxiety in shaping QoL outcomes. Moreover, while extensive research has been conducted worldwide on the factors associated with anxiety, depression, and QoL [13, 19, 20], such studies are notably limited in LMIC, specifically Indonesia. This gap is critical because the factors influencing mental health and QoL may differ in Indonesia due to varying socioeconomic conditions and the increasing burden of non-communicable diseases. Additionally, cultural differences in how mental health is perceived and addressed in the country, along with disparities in healthcare infrastructure, may result in distinct challenges that are not captured by research conducted in high-income countries. Given Indonesia's rising burden of mental health, understanding these mediation effects is crucial for designing effective interventions. This study is the first to apply Structural Equation Modelling (SEM) to a large population-based dataset in Indonesia to assess how depression and anxiety mediate the relationship between social and biological factors and QoL. Identifying these pathways provides critical insights for mental health policy and intervention strategies tailored to Indonesia's unique socio-demographic and healthcare landscape."

Comments from Reviewer #1

6. Ensure that the stated objectives align with the methodology and findings detailed later

Authors' response

We have ensured that the stated study aim aligns with the methodology and findings.

The objective of our study was to examine the association between social and biological factors and QoL and to assess whether depression and anxiety mediate this relationship.

To ensure this objective aligns with our methodology and findings, we structured our analysis using Structural Equation Modelling (SEM), a method commonly used in epidemiological research to explore complex interactions between multiple variables.

As illustrated in Figure 1, we conducted three models to systematically address our research questions:

1. Model 1: We examined the direct associations between social factors, biological factors, and QoL, addressing the first objective of the study.

2. Model 2 & Model 3: We introduced depression and anxiety as mediators, allowing us to assess whether these mental health conditions explain part of the relationship between social and biological factors and QoL. Our findings indicate that depression mediated the relationship between social and biological factors and QoL, confirming the mediation hypothesis outlined in our objective.

Thus, our methodological approach directly supports our study's objectives by first analysing the direct associations and then incorporating mediation effects through depression and anxiety.

Comments from Reviewer #1

Methods

7. Provide more detail about the randomization process and any potential biases in participant selection

Authors' response

We have added a paragraph detailing the randomisation process under the Sample Data subsection in the Method section (page 4):

"The randomisation process was conducted at the RT (neighborhood unit) level to ensure fair participant selection. First, the number of residents in each RT was determined using population data and verified through local authorities. The number of selected RTs was calculated using the formula 1,600 divided by the total population aged 18+ in each village, multiplied by 100%. RTs were then randomly chosen using STATA software to avoid selection bias, with three additional RTs selected as reserves to prevent sample shortages. All adults (18+) in the selected RTs were included as research participants. For the final RT, a random selection process ensured that every resident had an equal chance of participation."

Comments from Reviewer #1

8. Highlight the limitations of the CES-D scale's low Cronbach's alpha (0.566) and discuss its impact on the reliability of depression measurements

Authors' response

We have added the discussion on the limitations of the CES-D scale's low Cronbach's alpha in the Discussion section (page 11):

"Finally, this study has a relatively low internal consistency of the CES-D-10 scale (Cronbach's alpha=0.566). While this value is lower than the conventional threshold for strong reliability, it is still within an acceptable range for short screening tools, particularly in diverse population-based studies. The CES-D-10 has been widely used in mental health research, including in Indonesia, and remains a practical and efficient tool for detecting depressive symptoms in large-scale surveys. Although a lower Cronbach's alpha may indicate some variability in responses, our findings remain robust as they align with existing research on depression and quality of life."

Comments from Reviewer #1

9. While ethical approvals are noted, consider expanding on participant consent procedures to ensure clarity

Authors' response

We have added a paragraph detailing the participant consent procedure under the Sample Data subsection in the Method section (page 4 - 5):

"*In regard to participant consent, the procedure ensured that individuals had sufficient time to make an informed decision about their participation. Enumerators first visited potential participants' homes, provided them with a Participant Information Sheet (PIS), and allowed 24 hours for consideration. The following day, the enumerator returned to confirm their decision. If the individual agreed to participate, they were required to sign an informed consent form before the interview began, ensuring voluntary and ethical participation in the survey.*"

Comments from Reviewer #1

Discussion

10. Expand the discussion to compare findings with a broader range of international studies (e.g., 10.1186/s40359-023-01059-9)

Authors' response

Thank you for the feedback. We have expanded our discussion on the link between depression and quality of life in the LMICs (page 11):

"*Prior studies in LMICs revealed the relationships between depression and QoL [42, 43]. For example, Mohammadi et al. (2023) found that depression negatively affected both the physical (aOR = 0.966, p = 0.0186) and psychological (aOR=0.950, p=0.0005) domains of QoL among healthcare workers in Herat province, Afghanistan [43].*"

Comments from Reviewer #1

11. Elaborate on the theoretical pathways through which depression and anxiety mediate the effects of social and biological factors on QoL (e.g., DOI: DOI: 10.1093/pubmed/fdae111).

Authors' response

We have included the theoretical pathways to explain our findings on how biological conditions impact QoL through depression and anxiety, i.e., the biopsychosocial model in the Discussion section (page 10):

"*These findings are consistent with a biopsychosocial model [38, 39], which suggests that biological, psychological, and social factors influence QOL. Adopting this model, a study among multiple sclerosis patients found that beyond the physical symptoms, psychological factors such as depression and anxiety, along with social elements like support systems, play crucial roles in influencing QoL [39]. Another study used the biopsychosocial model to demonstrate that depression mediated the relationship between baseline pain catastrophising and the change in mental QoL after a year, highlighting the importance of psychological distress in explaining variations in well-being among patients with long-term illnesses [40].*"

Comments from Reviewer #1

12. Discuss potential biases, including self-reporting and the cross-sectional design, in greater detail

Authors' response

We have added one literature which discussed about the potential bias from self-reporting and cross-sectional design in page 11.

'*The cross-sectional design prevents us from establishing causal relationships between variables, as it captures data at a single point in time. Future research should implement longitudinal designs to understand better the temporal sequencing and directionality of the relationships between biological and social factors, mental health, and QoL. To address this limitation, we plan to collect a second wave of data in 2025, allowing for a longitudinal follow-up analysis, which will be the first longitudinal study focusing on mental health, risk factors, and healthcare utilisation in Indonesia. This will enable us to*

assess changes in depression, anxiety, and QoL over time, providing stronger evidence for mediation effects and long-term intervention implications. Additionally, the information on comorbidities was self-reported, which could introduce biases such as recall inaccuracies or social desirability effects, potentially compromising the accuracy of the findings [45]. Similarly, self-reported health conditions might be influenced by underdiagnosis or selective reporting. Therefore, caution is advised when interpreting the results concerning the biological factors, and future studies should aim to incorporate more objective measures to strengthen the robustness of their conclusions.'

Comments from Reviewer #1

13. Emphasize specific recommendations for interventions in LMICs, tailored to the Indonesian context

Authors' response

We have revised the Conclusion section and include the recommendations for intervention tailored to the Indonesia context (Page 12):

"*This study found that depression and anxiety significantly mediate the relationship between social and biological factors and QoL in Indonesia. To improve mental health and QoL in Indonesia, proactive promotion and prevention strategies must be implemented at individual and community levels. Public health initiatives should focus on enhancing physical and social well-being by promoting healthy lifestyles, encouraging regular physical activity, and improving nutrition through government-supported wellness programs. Additionally, strengthening social networks is crucial, particularly in rural and low-resource settings where access to mental health care is limited. Social isolation is a known risk factor for poor mental health outcomes, and interventions aimed at enhancing social connections have been shown to improve well-being, engagement in care, and quality of life among individuals with mental health conditions [46]. Community-based interventions, such as leveraging Posyandu (integrated health posts) to deliver psychoeducation, stress management workshops, and peer support groups, can foster social connections and reduce the stigma around mental health.*

Given that depression and anxiety were only screened in this study, early intervention programs—such as scaling up mental health screening at Puskesmas (community health centres) and integrating psychological support into Indonesia's National Health Insurance Scheme (BPJS)—could prevent worsening conditions. Expanding digital mental health platforms, including SMS-based counselling and telepsychiatry services, can help reach underserved populations. Integrating these strategies within primary care, schools, and workplaces ensures a holistic and sustainable approach to improving mental health and QoL in Indonesia."

Comments from Reviewer #1

Conclusion

14. Condense the conclusion to focus on the most critical findings and their implications

Authors' response

We have condensed the conclusion to provide a more concise and focused summary of the key findings and add the implications tailored for the Indonesia context (Page 12).

'*This study found that depression and anxiety significantly mediate the relationship between social and biological factors and QoL in Indonesia. To improve mental health and QoL in Indonesia, proactive promotion and prevention strategies must be implemented at individual and community levels. Public*

health initiatives should focus on enhancing physical and social well-being by promoting healthy lifestyles, encouraging regular physical activity, and improving nutrition through government-supported wellness programs. Additionally, strengthening social networks is crucial, particularly in rural and low-resource settings where access to mental health care is limited. Social isolation is a known risk factor for poor mental health outcomes, and interventions aimed at enhancing social connections have been shown to improve well-being, engagement in care, and quality of life among individuals with mental health conditions [46]. Community-based interventions, such as leveraging Posyandu (integrated health posts) to deliver psychoeducation, stress management workshops, and peer support groups, can foster social connections and reduce the stigma around mental health.

Given that depression and anxiety were only screened in this study, early intervention programs—such as scaling up mental health screening at Puskesmas (community health centres) and integrating psychological support into Indonesia's National Health Insurance Scheme (BPJS)—could prevent worsening conditions. Expanding digital mental health platforms, including SMS-based counselling and telepsychiatry services, can help reach underserved populations. Integrating these strategies within primary care, schools, and workplaces ensures a holistic and sustainable approach to improving mental health and QoL in Indonesia.'

<u>Comments from Reviewer #1</u>

15. Provide a more detailed outline of proposed longitudinal studies or interventions to address the identified research gaps

<u>Authors' response</u>

We have added the detailed outline of the proposed longitudinal study in Page 11 and interventions in page 12 (please see above response for the interventions):

"*The cross-sectional design prevents us from establishing causal relationships between variables, as it captures data at a single point in time. Future research should implement longitudinal designs to understand better the temporal sequencing and directionality of the relationships between biological and social factors, mental health, and QoL. To address this limitation, we plan to collect a second wave of data in 2025, allowing for a longitudinal follow-up analysis, which will be the first longitudinal study focusing on mental health, risk factors, and healthcare utilisation in Indonesia.*"

<u>Comments from Reviewer #2</u>

Abstract

1. The methods section—describe the methods only, results (including number of participants and mean age) should not be included

<u>Authors' response</u>

The abstract section has been revised in response to the reviewer's comment to describe only the methods, with the number of participants and mean age removed:

"*Methods: This study used data from a large cross-sectional household survey in Indonesia to examine individuals aged 18 and older. Depression and anxiety were measured using the CES-D-10 and GAD-7 scales, while QoL was assessed using the EQ-5D questionnaire. Social factors were assessed using the self-reported number of close persons, and biological factors were measured using the number of self-reported physical health comorbidities. Structural Equation Modelling (SEM) was used to explore these relationships. We adjusted the models by age and sex*."

Comments from Reviewer #2

Introduction

2 Page 3, line 53 – 55: the authors should elaborate more on the connection between social and biological factors and QoL—with reference to extant literature and what the gaps in the literature are. As it stands there is no clear rationale for the study

Authors' response

We have revised the introduction to provide a more detailed explanation of how social and biological factors influence mental health and, in turn, how these two factors affect quality of life. Additionally, we have explicitly highlighted the gap in the existing literature, emphasising the need for further research, particularly in the context of low- and middle-income countries (LMICs) like Indonesia:

"*Prior studies have demonstrated the importance of these factors for QoL. However, whether depression and anxiety mediate the link between social and biological factors and QoL is not yet known. Most existing research focuses on social and biological factors as the determinants of QoL, leaving a significant gap in understanding the mechanisms explaining the relationships between those two variables [7, 9, 10, 13, 18]. Most existing studies focus on direct associations, without considering the mediating role of depression and anxiety in shaping QoL outcomes. Moreover, while extensive research has been conducted worldwide on the factors associated with anxiety, depression, and QoL [13, 19, 20], such studies are notably limited in LMIC, specifically Indonesia. This gap is critical because the factors influencing mental health and QoL may differ in Indonesia due to varying socioeconomic conditions and the increasing burden of non-communicable diseases. Additionally, cultural differences in how mental health is perceived and addressed in the country, along with disparities in healthcare infrastructure, may result in distinct challenges that are not captured by research conducted in high-income countries. Given Indonesia's rising burden of mental health, understanding these mediation effects is crucial for designing effective interventions. This study is the first to apply Structural Equation Modelling (SEM) to a large population-based dataset in Indonesia to assess how depression and anxiety mediate the relationship between social and biological factors and QoL. Identifying these pathways provides critical insights for mental health policy and intervention strategies tailored to Indonesia's unique socio-demographic and healthcare landscape."*

Comments from Reviewer #2

3. The authors mentioned social and biological factors influencing quality of life but did not fully explore this. What are these social and biological factors? Has existing literature found similar associations between these factors and anxiety and depression? any existing theories or works that have suggested that anxiety and depression may mediate the relationship between social and biological factors and quality of life?

Authors' response

We have revised the manuscript to provide a more comprehensive discussion of the social and biological factors examined in our study and their relationship with anxiety, depression, and QoL by explicitly defining the social and biological factors analysed in the existing literature, and how prior research has linked these variables to mental health and QoL (Page 3):

"*Depression and anxiety are complex conditions with multiple causes, including social and biological factors. Social factors are crucial to mental health, as shown by two systematic reviews that found a*

strong link between protective social factors and a lower likelihood of depression [5], as well as reduced severity of anxiety [6]. Biological factors also significantly affect quality of life (QoL), as poor self-rated health, abnormal body mass index (BMI), and the presence of chronic illnesses have been strongly associated with higher levels of depression [7] and anxiety [8]. Furthermore, prior studies have shown that both social and biological factors affect QoL [9, 10]. For example, a study in Ethiopia found that social support level and duration of illness were significant predictors of QoL [9]. Additionally, a study conducted in Iran, another LMIC, also found that higher perceived social support and not having any chronic diseases were associated with higher QoL [10]. These relationships suggest that social and biological factors and QoL may be influenced either directly or indirectly by the presence of depression and anxiety."

Comments from Reviewer #2

Methods

a. Sample Data:

4. The authors sampled 12 villages – 6 rural and 6 urban. It is not clear how these 12 villages were chosen. How did the author avoid selection bias?

Authors' response

This concern was previously addressed, and we have already added a detailed explanation of the randomisation process under the Method, Sample Data section (pages 3–4).

To summarise, RTs (neighborhood units) were randomly selected using STATA software based on verified population data, ensuring fair participant selection and minimising selection bias. Reserve RTs were also chosen to account for potential sample shortages, and all adults (18+) in the selected RTs were included. For the final RT, a further randomisation process ensured equal participation opportunities.

Comments from Reviewer #2

5. Given the difference in population between rural and urban villages, a proportionate sampling technique should have been employed. This will ensure that the sample will be representative of the population.

Authors' response

Thank you for the comments. In our study, proportionate stratified sampling was not applied, as the sampling strategy was designed to balance geographical diversity and feasibility while ensuring a sufficient sample size across locations. Specifically, data collection occurred from July 27 to October 20, 2023, across 12 villages in 6 districts/cities within four provinces on Java Island, purposefully selected based on urban and rural characteristics, population size, density, and the availability of primary health centres. Six villages were chosen to represent urban areas, and six represented rural areas.

Randomisation was conducted at the RT (neighbourhood unit) level to ensure fair participant selection. Instead of proportionate sampling, we used a randomised RT selection process, verified through local population data and authorities. The number of RTs sampled in each village was determined using a formula based on the total number of residents aged 18+ to ensure adequate coverage within each village. This method minimised selection bias while ensuring that urban and rural differences were

captured in a structured and systematic way. Although our approach does not apply proportionate weighting, it ensures that both rural and urban populations were adequately represented through randomised selection within each village. Future studies may consider weighting adjustments or stratified random sampling to achieve closer proportional representation of Indonesia's broader demographic landscape.

To enhance clarity, we have revised the Methods and Limitation sections of the manuscript to acknowledge this consideration and provide additional details on our sampling approach.

"Another limitation is that we employed a structured randomisation process at the RT (neighbourhood unit) level, ensuring fair selection within each village. Although this method minimised selection bias, the population distribution between urban and rural settings was not strictly proportionate. Future studies should consider proportionate weighting or stratified random sampling to enhance demographic representativeness."

Comments from Reviewer #2

Measures

6. Social factors: why did the author choose social network as the sole social factor to identify. Is this based on literature review?

Authors' response

Yes, we choose the social network as the social factor based on the literature review. We have added the literature supporting our method (page 5):

"*The social factor examined in this study is the individual's social network, measured by the number of people with whom participants reported they frequently discussed important matters. This measure has been used in LMICs to identify the link between social factors and late-life depression in LMICs [21, 22]. A review, including studies from 16 LMICs, found that larger social networks, characterised by a higher number of close contacts, are indeed protective against depression among older adults [23]. A study in Aceh, Indonesia, revealed that the number of close relationships has the most significant relationship with individuals' mental health and overall well-being [22].*"

Comments from Reviewer #2

7. Also, there are standardized measures that have been validated for measuring social network; why did the authors not use those?

Authors' response

We appreciate the reviewer's suggestion regarding the use of standardised measures for assessing social networks. Our study used the number of the people close with the respondents as the measure due to several key considerations:

- Context-specific relevance: Many standardised social network measures were developed in Western contexts and may not fully capture social dynamics in Indonesia, where family ties, religious groups, and community engagement play a unique role in social support.
- Feasibility and participant burden: Standardised social network instruments often involve detailed and time-intensive questionnaires. Given the large-scale, community-based data collection in this study, using a shorter, structured measure was necessary to ensure participant engagement and response quality.

- Alignment with research focus: Our study focused on the role of social network size and perceived support in mental health and QoL. The measure used was informed by previous studies and tailored to capture key social relationships relevant to mental well-being in Indonesia.
- Study design considerations: Given the cross-sectional design and survey constraints, we prioritised a concise but meaningful assessment of social connections, ensuring that the measure was practical for use in large-scale data collection.

We have now clarified this rationale in the Measures section of the manuscript.

"*The social factor examined in this study is the individual's social network, measured by the number of people with whom participants reported they frequently discussed important matters. This measure has been used in LMICs to identify the link between social factors and late-life depression in LMICs [21, 22]. A review, including studies from 16 LMICs, found that larger social networks, characterised by a higher number of close contacts, are indeed protective against depression among older adults [23]. A study in Aceh, Indonesia, revealed that the number of close relationships has the most significant relationship with individuals' mental health and overall well-being [22].*"

<u>Comments from Reviewer #2</u>

8. Social network is a complex construct, it is unlikely that one question would measure the construct. Therefore, the validity of the construct is questionable.

<u>Authors' response</u>

We have included the literature supporting our method – Measures section (page 5 - 6).

<u>Comments from Reviewer #2</u>

9. Also, it is unclear how a Likert scale with 8 responses was generated for the social network question. The phrasing is most suitable for a yes or no answer. The validity of the construct is questionable.

<u>Authors' response</u>

We appreciate the reviewer's comment regarding the format of the social network question. We clarify that the eight-response scale does not represent a Likert scale but rather a numerical count of the number of close social ties. We have revised the Measures section to explain the method better:

"*To capture this, we asked the respondents, "Are there other people with whom you often discuss things?". If the respondent answered "yes", we repeated this question six more times to determine the number of close social ties they identified. The final measure was obtained by summing these responses, resulting in a range from 0 to 7. For analysis, we treated social network size as a continuous variable, where higher values indicate a larger social network*."

<u>Comments from Reviewer #2</u>

10. Biological factors: recall bias. What about access to care—if these conditions are present but not diagnosed. Also, it is not clear what the authors mean by "the number ranges between 0 and 10". Does this mean that each condition is given a score of 1? Also, there are a lot of chronic conditions, how did

the authors settle on the ones included in the survey? Also, ‘other lung diseases’ could mean a host of conditions from acute to chronic lung diseases—were the authors specific on the chronicity?

Authors’ response

We acknowledge that self-reported chronic conditions may be subject to recall bias, as participants may not accurately remember past diagnoses. We have included this limitation in the Discussion section:

“*Additionally, the information on comorbidities was self-reported, which could introduce biases such as recall inaccuracies or social desirability effects, potentially compromising the accuracy of the findings [45]. Similarly, self-reported health conditions might be influenced by underdiagnosis or selective reporting. Therefore, caution is advised when interpreting the results concerning the biological factors, and future studies should aim to incorporate more objective measures to strengthen the robustness of their conclusions.*”

We included the information that the comorbidities included in our study were based on prior Indonesian national surveys. We have also added the definition of other lung diseases in this study (page 5):

“*These conditions were chosen based on their inclusion in prior national health surveys in Indonesia, such as the Indonesian Family Life Survey (IFLS) [24] and the Indonesia National Basic Health Survey (Riset Kesehatan Dasar or Riskesdas) [25], which have consistently used similar categories to assess disease burden in the population. The category “other lung diseases” was included to capture any additional chronic respiratory conditions not specifically listed, such as chronic obstructive pulmonary diseases, chronic bronchitis, emphysema, or other persistent pulmonary diseases*.”

Comments from Reviewer #2

11. Quality of life measure: why did the author use the Great Britian value? Also, was this instrument translated into the local language?

Authors’ response

The EQ-5D is one of the most used generic health-related quality-of-life (HRQOL) instruments worldwide. It has been validated in Indonesia and is widely used in health research. We used the Bahasa Indonesia version of the EQ-5D (ref). We used the GB value set based on practical constraints, as the STATA command for computing health utility values (e.g., the eq5d command) provides pre-programmed value sets for only three countries: Great Britain, the Netherlands, and Spain. Among these available options, GB was selected as the closest match for our study because it aligns with commonly used international value sets in health economic evaluations and has been widely applied in global research. Furthermore, among the three available value sets, the GB value set provides a more generalisable approximation for an LMIC setting compared to the Netherlands or Spain, based on previous studies using similar approaches.

Ref:

Prabowo, Muhammad Husen, et al. "Health-related quality of life of patients with Diabetes Mellitus measured with the Bahasa Indonesia Version of EQ-5D in primary care settings in Indonesia." Journal of Preventive Medicine and Public Health 56.5 (2023): 467.

Purba FD, Hunfeld JAM, Iskandarsyah A, Fitriana TS, Sadarjoen SS, Ramos-Goñi JM, Passchier J, Busschbach JJV. The Indonesian EQ-5D-5L Value Set. Pharmacoeconomics. 2017 Nov;35(11):1153-1165. doi: 10.1007/s40273-017-0538-9. PMID: 28695543; PMCID: PMC5656740.

Comments from Reviewer #2

Mediating Factors

12. CES-D-10: 'The reliability test, with a Cronbach's alpha of 0.566, and the model fit for CFA are detailed in Supplementary 1 and 2A."—did the authors conduct a validation study on the CES-D? If yes, was this part of the current study? did they use the same sample as for the main study?

Authors' response

To clarify, we did not conduct a separate validation study on the CES-D-10 as part of this research. However, we performed a reliability analysis (Cronbach's alpha = 0.566) and a confirmatory factor analysis (CFA) to assess the scale's internal consistency and model fit within our study sample. These analyses were conducted using the same dataset as the main study, and the results are provided in Supplementary 1 and 2A.

We clarify this in the Methods section (page 6):

"The Indonesia version of CESD-10 has further shown strong test-retest reliability and internal consistency, with an interclass correlation of 0.85 and a Cronbach's α of 0.86 among individuals aged 15 and older [24] and 0.9 among adolescents [30]. The Cronbach's α for this study is 0.566 (Supplementary 1 and 2A)."

Comments from Reviewer #2

13. Similar question for the GAD-7: the reported Cronbach's alpha, was this part of the current study and using the same sample?

Authors' response

Similar to the CES-D-10, we did not conduct a separate validation study for the GAD-7 but performed a reliability test (Cronbach's alpha) and confirmatory factor analysis (CFA) as part of this research. These analyses were conducted using the same dataset as the main study, and the results are presented in Supplementary 1 and 2B.

We clarify this in the Methods section (page 6):

"A prior study has validated GAD-7 among Indonesians and showed good internal validity and reliability with a Cronbach's α score of 0.867 and a validity coefficient ranging from 0.648 to 0.800 (p<0.01) [32]. The reliability test of GAD-7 in this study, indicated by a Cronbach's α of 0.770, and the model fit for CFA are detailed in Supplementary 1 and 2B."

Comments from Reviewer #2

Statistical Analysis

14. "We summarised the participants' characteristics by calculating the mean and standard deviation (SD) for continuous variables and reporting counts and percentage for categorical variables. To

compare these variables between males and females, we utilised the chi-square test for nominal and ordinal variables and the Kruskal-Wallis test for continuous variables"—

The Kruskal-Wallis test assumes that the data are non-parametric, meaning they do not follow a normal distribution. Therefore, it would be more appropriate to report the median and interquartile range rather than the mean and standard deviation as measures of central tendency and variability, as the latter may not accurately represent the data distribution.

Authors' response

The continuous data has been replaced with the median and interquartile range (Table 1), as these are more appropriate measures of central tendency and variability for non-parametric data in the Kruskal-Wallis test.

Comments from Reviewer #2

Discussion

15. Page 9, line 34 – 50. The authors discuss how comorbidities impact quality of life by citing factors such as loss of productivity, increased healthcare expenditure, and more frequent hospital visits. While loss of productivity and increased healthcare spending can be considered social factors that may mediate the relationship between comorbidities and quality of life, this study focuses solely on social networks. As a result, the model may not fully capture the broader influence of social determinants on quality of life, potentially limiting the generalizability of the findings

Authors' response

In our discussion, we highlighted factors such as loss of productivity, increased healthcare expenditure, and more frequent hospital visits as potential mechanisms through which comorbidities may impact QoL. However, we acknowledge that loss of productivity and financial burden are broader social determinants of health that may act as additional mediators beyond the social network variable examined in our study. Given that our analysis specifically focused on social network size as the primary social factor, the model does not capture the full scope of socioeconomic pathways through which comorbidities influence QoL.

To address this, we have revised the Discussion section to acknowledge this limitation explicitly. We now clarify that future research should consider incorporating additional social determinants—such as economic hardship, access to healthcare, and employment status—to provide a more comprehensive understanding of the relationship between multimorbidity and QoL.

Comments from Reviewer #2

16. Page 9, lines 53- 57: "Specifically, having more comorbidities was associated with increased levels of anxiety and depression, suggesting that the emotional and physical burden of managing multiple chronic conditions, including changes in lifestyle, contributes to increased psychological distress"—citation needed

Authors' response

We have included the citations of the sentences. – [37]

Ref:

Felez-Nobrega M, Haro JM, Koyanagi A. Multimorbidity, depression with anxiety symptoms, and decrements in health in 47 low- and middle-income countries. Journal of Affective Disorders. 2022;317:176-84.

Comments from Reviewer #2

17. Page 10, lines 29 – 36: The authors assert that depression and anxiety fully mediate the relationship between social factors and QoL. While their model suggests full mediation, the interpretation should acknowledge that statistical mediation does not imply causality. The cross-sectional nature of the study limits the ability to establish directionality, and unmeasured confounders or alternative pathways may still exist. Additionally, the statement that "increasing levels of depression and anxiety lead to a reduction in QoL" implies a causal effect, which is not supported by mediation analysis alone. A more cautious interpretation should emphasize that depression and anxiety statistically account for the relationship rather than determining it exclusively. The authors should also clarify whether model fit and potential confounding variables were adequately addressed to support the claim of full mediation.

Authors' response

We recognise that statistical mediation does not imply causality, especially given the cross-sectional nature of the study, which limits the ability to establish directionality between social factors, mental health, and quality of life (QoL). While our model suggests that depression and anxiety fully mediate the relationship between social factors and QoL, this should be understood as a statistical association rather than a causal determination. Unmeasured confounders or alternative pathways may also influence this relationship, which should be considered when interpreting the results.

To ensure greater clarity, we have revised the Results and Discussion sections to reflect that depression and anxiety statistically account for the relationship between social factors and QoL, rather than exclusively determining it. Additionally, the phrase "increasing levels of depression and anxiety lead to a reduction in QoL" has been modified to "increasing levels of depression and anxiety are related to a lower QoL."

Regarding model fit and confounding variables, we confirm that:

- We tested model fit using [state which fit indices were used, e.g., CFI, RMSEA, SRMR], and the results indicated acceptable model fit, as detailed in Supplementary 2C.
- Potential confounders, such as age, gender, and socioeconomic status, were controlled for in the mediation model to reduce bias. However, we acknowledge that residual confounding from unmeasured variables remains a limitation, and we recommend future longitudinal studies to establish clearer directionality.

Comments from Reviewer #2

18. Page 10, line 38 – 49: The statement overstates the findings by implying that social factors impact QoL entirely through depression and anxiety, especially considering that only one social factor—social network—was examined in the study. Mediation analysis demonstrates statistical relationships but does not establish causality or preclude other direct or indirect pathways. The authors should adopt more cautious language to reflect the observational nature of the study. Furthermore, while the discussion of biological mechanisms is relevant, its connection to QoL remains unclear, as it does not explicitly explain how inflammation directly contributes to reduced quality of life.

Authors' response:

We have revised the statement on page 10, lines 38-49, to avoid the causality. We removed the explanation of how inflammation directly contributes to reduced quality of life from the Discussion section.

Comments from Reviewer #2

Study Strengths

19. Page 10, lines 50 -60: The study's large sample size enhances statistical power, but generalizability claims should be made cautiously. The selection of 12 villages—6 urban and 6 rural—without clear selection criteria raises concerns about representativeness. The reported "randomised sampling method" requires clarification, as the equal allocation of participants may not reflect actual population distributions as rural and urban populations differ in size. Proportionate sampling would improve representativeness and reduce bias. The claim of providing a "nuanced understanding" should be framed more cautiously to reflect the study's observational nature.

Authors' response

We have revised the study strengths section:

"*This study exhibits several key strengths. Firstly, it includes a large population cohort of over 19,000 participants, providing robust statistical power and generalisability in urban and rural communities in the four provinces in Java, Indonesia*."

Comments from Reviewer #2

Supplementary materials

20. It remains unclear whether the CFA results shown are for the present study or reported from another study. If from present study, was this a pilot or part of the main study?

Authors' response

We clarify that the CFA was conducted as part of the present study using the same sample as the main analysis. It was not a pilot study, nor were the results taken from another study.

The CFA was performed to assess the factor structure and model fit of the CES-D-10 and GAD-7 within our sample. The results, including fit indices such as CFI, RMSEA, and SRMR, are reported in Supplementary 2A and 2B. This analysis was conducted to ensure that the scales retained their original structure and were suitable for use in this population, given that validated psychometric properties in Indonesia are limited.

To enhance clarity, we have revised the Measures and Supplementary Materials sections to explicitly state that the CFA was conducted within this study and using the full study sample.

Comments from Reviewer #2

21. If previous validation studies on the GAD-7 and CES-D have been conducted, they should be included in the main manuscript as part of the survey instrument description, rather than in the supplementary section.

Authors' response

We have added prior studies validated GAD-7 and CESD-10 in Indonesia in the Method section:

"The Indonesia version of CESD-10 has further shown strong test-retest reliability and internal consistency, with an interclass correlation of 0.85 and a Cronbach's α of 0.86 among individuals aged 15 and older [24] and 0.9 among adolescents [30]."

"A prior study has validated GAD-7 among Indonesians and showed good internal validity and reliability with a Cronbach's α score of 0.867 and a validity coefficient ranging from 0.648 to 0.800 (p<0.01) [32]."

VERSION 2 - REVIEW

**Reviewer**	**1**
**Name**	**Neyazi, Ahmad**
**Affiliation**	**Afghanistan Center for Epidemiological Studies**
**Date**	**17-Mar-2025**
**COI**	

I would like to extend my appreciation to the authors for thoroughly addressing all the comments and suggestions provided. The revisions have significantly improved the manuscript, and in its current form, I believe it meets the necessary standards for publication in BMJ Open.

**Reviewer**	**2**
**Name**	**Ogundare, Temitope**
**Affiliation**	**Boston University**
**Date**	**27-Mar-2025**
**COI**	

All comments addressed
